# Relationships between triglyceride-glucose index and incident gestational diabetes mellitus: a prospective cohort study of a Korean population using publicly available data

**DOI:** 10.3389/fpubh.2024.1294588

**Published:** 2024-02-13

**Authors:** Zihe Mo, Changchun Cao, Yong Han, Haofei Hu, Yongcheng He, Xin Zuo

**Affiliations:** ^1^Department of Physical Examination, DongGuan Tungwah Hospital, Dongguan, Guangdong, China; ^2^Department of Rehabilitation, Shenzhen Dapeng New District Nan'ao People's Hospital, Shenzhen, Guangdong, China; ^3^Department of Emergency, Shenzhen Second People's Hospital and The First Affiliated Hospital of Shenzhen University, Shenzhen, Guangdong, China; ^4^Department of Nephrology, Shenzhen Second People's Hospital and The First Affiliated Hospital of Shenzhen University, Shenzhen, Guangdong, China; ^5^Department of Nephrology, Shenzhen Hengsheng Hospital, Shenzhen, Guangdong, China; ^6^Department of Nephrology, Affiliated Hospital of North Sichuan Medical College, Nanchong, Sichuan, China; ^7^Department of Endocrinology, Shenzhen Third People's Hospital, Shenzhen, Guangdong, China

**Keywords:** triglyceride-glucose index, triglyceride, gestational diabetes mellitus, logistic regression model, insulin resistance, receiver operating characteristic curve

## Abstract

**Background:**

The connection between the triglyceride-glucose index (TyG index) and gestational diabetes mellitus (GDM) is currently debated. Our study aimed to investigate the connection between the TyG index and GDM within the Korean population.

**Methods:**

Using publically accessible data in Korea, we performed a secondary study on a sample of 589 pregnant women who were carrying a single fetus. The analysis employed a binary logistic regression model, some sensitivity analyses, and subgroup analysis to investigate the association between the TyG index and the occurrence of GDM. To assess the TyG index’s potential to predict GDM, a receiver operating characteristic (ROC) study was also carried out.

**Results:**

The mean age of the pregnant women was 32.065 ± 3.798 years old, while the mean TyG index was 8.352 ± 0.400. The prevalence rate of GDM was found to be 6.112%. Upon adjusting for potential confounding variables, a positive association was detected between the TyG index and incident GDM (OR = 12.923, 95%CI: 3.581–46.632, *p* = 0.00009). The validity of this connection was further confirmed by subgroup analysis and sensitivity analyses. With an area under the ROC curve of 0.807 (95%CI: 0.734–0.879), the TyG index showed strong predictive power for GDM. The TyG index’s ideal cutoff value for detecting GDM was found to be 8.632, with a sensitivity of 78.7% and a specificity of 72.2%.

**Conclusion:**

The findings of our study provide evidence that an increased TyG index is significantly associated with the occurrence of GDM. Utilizing the TyG index during the 10–14 week gestational period may be a valuable tool in identifying pregnant individuals at a heightened risk for developing GDM. Early detection enables timely and efficacious interventions, thereby enhancing the prognosis of affected individuals.

## Introduction

Gestational diabetes mellitus (GDM) refers to varying degrees of glucose intolerance that occur or are identified for the first time during pregnancy, irrespective of pre-existing diabetes (1). During pregnancy, GDM is a prevalent complication, with its incidence steadily rising in recent decades (2–4). The etiology of GDM is multifaceted, encompassing obesity/pre-gravidic weight, maternal age, and history of polycystic ovary syndrome (5, 6). Notably, GDM is associated with an increased likelihood of adverse perinatal outcomes, such as pre-eclampsia, gestational hypertension, miscarriage, cesarean section, and macrosomia (2, 7, 8). Furthermore, GDM has been acknowledged as a significant predisposing factor for maternal cardiovascular disease and diabetes (9), as well as obesity and insulin resistance (IR) in the offspring (10). The conventional approach for the clinical ascertainment of GDM is conducted within the 24–28th weeks of gestation, employing a 75 g oral glucose tolerance test (OGTT) delineated in the literature (11). However, empirical evidence suggests that by the time GDM is diagnosed at this stage, both the mother and fetus may have already been adversely affected to varying degrees despite the potential benefits of symptom management (12, 13). Therefore, the timely recognition of women at heightened risk for gestational diabetes mellitus is of paramount importance for mitigating the potential adverse outcomes and stemming the tide of transgenerational metabolic sequelae.

Previous studies have indicated that insulin resistance (IR) is a critical element in both the onset and progression of GDM. It is characterized by an impaired response to insulin in peripheral tissues, which becomes particularly problematic during pregnancy as the demand for insulin escalates (14). The insidious nature of IR often means that it is well established by the time GDM is clinically recognized, contributing to the challenge of timely diagnosis and management (15). The interaction between maternal IR and β-cell dysfunction is a central component in the pathophysiology of GDM (16). However, there is a scarcity of previous studies examining the potential predictive value of IR for GDM; previous studies likely lack a dependable and practical surrogate marker for IR (17). Traditionally, the definitive test for insulin sensitivity is the hyperinsulinemic-euglycemic clamp test (18). Nevertheless, this method is time-consuming and expensive, significantly limiting its use in clinical practice (19). In recent research, the triglyceride-glucose index (TyG index), a metric generated from fasting blood glucose and triglyceride levels, has been recommended as a trustworthy and practical diagnostic of IR (20, 21). A greater TyG index has been linked to a higher risk of type 2 diabetes mellitus (T2DM) in the adult population, according to prior studies (22). Based on the results of earlier investigations, it is not yet obvious if the TyG index can predict the risk of GDM (23–26). Consequently, the objective of this investigation was to comprehensively assess the prospective predictive ability of the TyG index for GDM within a cohort study of the Korean population, utilizing publicly available data.

## Methods

### Data source

The primary data utilized in this research were generously provided by Lee SM et al. (27). The primary data are available to the public. They are published under the Creative Commons Attribution License, which allows free use, distribution, and reproduction in any format as long as the author and source are properly acknowledged. We express our gratitude to the data contributors for their invaluable contributions.

### Study population

Between November 2014 and July 2016, the initial study encompassed 663 singleton pregnant women who had sought antenatal care at two prominent medical institutions, namely the Incheon Seoul Women Hospital and Seoul Metropolitan Government Seoul National University Boramae Medical Center, both located in Seoul, Korea. These participants were included if they had commenced prenatal care before reaching 14 weeks gestation. These women were recruited within the ongoing “Fatty Liver in Pregnancy” registry framework. Notably, before their inclusion, all singleton pregnant women provided written informed consent (27). The original professional staff employed a comprehensive and non-selective approach to meticulously collect cases for the original study.

The research ethics of this study were approved by the committee of the Seoul Metropolitan Government Seoul National University Boramae Medical Center and the committee of the Ministry of Health and Welfare of Korea (27). Therefore, given this prior ethical approval, no additional ethical clearance was required for this secondary analysis. Additionally, the primary research complied with the principles outlined in the Declaration of Helsinki.

**Figure 1:**
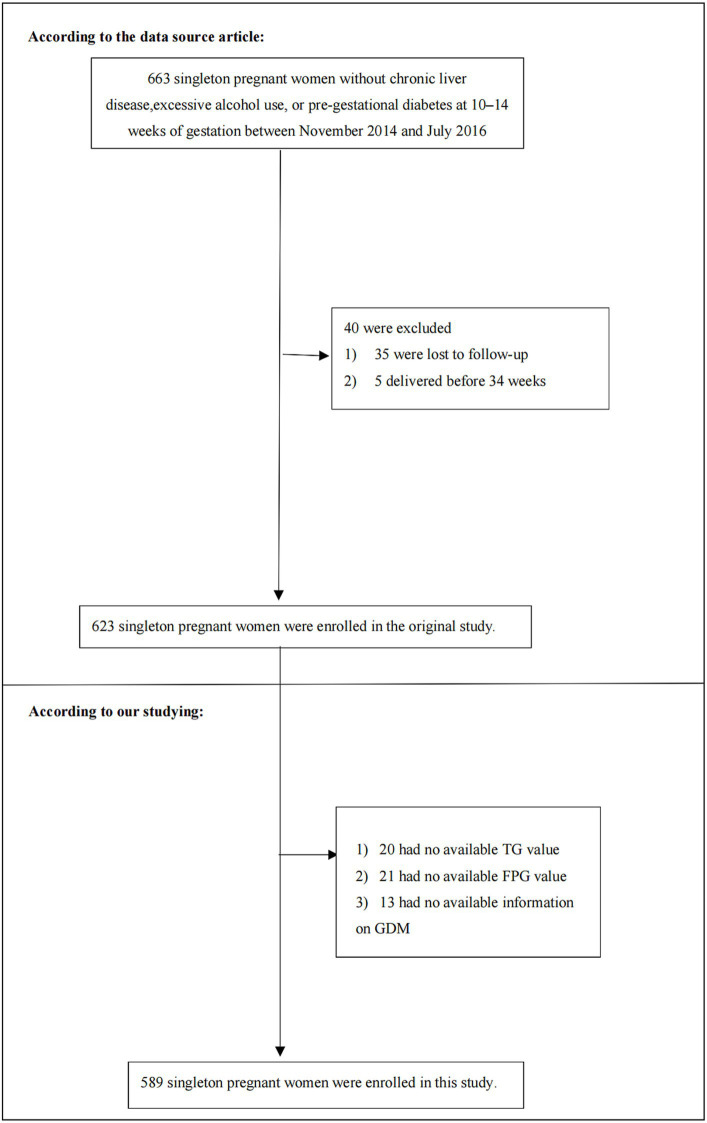
Flowchart of study participants. Figure showed the inclusion of participants. Six hundred and twenty-three participants were assessed for eligibility in the original study. We excluded patients with missing values of FPG (*n* = 21), TG (*n* = 20), GDM (*n* = 13). The final analysis included 589 subjects in the present study.

For the initial study, patients were excluded if they had (1) previous diagnosis of GDM, high alcohol consumption (more than 20 grams of alcohol per day), or chronic liver disease; (2) preterm delivery occurring before 34 weeks; or (3) were lost to follow-up. As a result, the initial study comprised 623 participants. Subsequently, we further excluded participants with missing data for GDM (*n* = 13), fasting plasma glucose (FPG) (*n* = 21), and triglyceride (TG) (*n* = 20). The final analysis included 589 singleton pregnant women. Figure 1 in the manuscript illustrates the study’s design and the flow of participants.

### Variables

#### TyG index

Venous blood samples from the subjects were taken between 10 and 14 weeks of pregnancy following a minimum 8-h fast. These specimens were subsequently subjected to centrifugation at an acceleration of 2000 *g* for a temporal span of 10 min, then partitioned into aliquots for preservation at a temperature of −70°C until the assay could be conducted. The intra-coefficient variation and inter-coefficient variation for FPG measured with the Roche/Hitachi 911 chemistry analyzer (Roche Diagnostics) were 1.75 and 2.33%, respectively. Similarly, the intra-coefficient variation and inter-coefficient variation for TG using the same analyzer were 3.50 and 4.66%, respectively. The precise method for calculating the TyG index is Ln[(TG (mg/dL) × FPG (mg/dL)/2)] (28).

#### Diagnosis of incident GDM

All participants were diagnosed with GDM in the two-step method during 24–28 weeks (27). For the initial screening, serum glucose levels were measured after a non-fasting 50 g oral glucose challenge (GCT) test, taken 1 h after consuming a 50 g oral glucose load. A blood glucose level of ≥7.8 mmol/L indicated a positive result on the GCT. An additional 100 g OGTT was administered to people who had a positive result on the GCT. GDM was established when two or more glucose levels were elevated: FPG ≥5.3 mmol/L, one-hour glucose ≥10 mmol/L, two-hour glucose ≥8.6 mmol/L, and three-hour glucose ≥7.8 mmol/L.

#### Covariates

In selecting risk variables for this study, a comprehensive approach was undertaken, drawing insights from clinical expertise, the original research, and existing literature on risk factors associated with GDM. Therefore, based on the above considerations, the following variables were adopted as covariates: (1) continuous variables: high-density lipoprotein cholesterol (HDL-C), age, insulin, aspartate aminotransferase (AST), low-density lipoprotein cholesterol (LDL-C), alanine aminotransferase (ALT), pre-pregnancy body mass index (BMI), total cholesterol (TC), gamma-glutamyl transferase (GGT); (2) categorical variables: parity, hepatic steatosis.

The general clinical and demographic data collection encompassed maternal age, prior history of GDM, height, parity, and pre-gestational weight. These details were gathered using a standardized questionnaire. Venous blood samples were obtained during the 10–14 weeks of pregnancy, ensuring an 8-h fasting period, to assess hematological markers, including GGT, TG, ALT, insulin, FPG, TC, and AST levels. Hepatic steatosis severity was determined using a previously established semiquantitative grading system (grades 0–3) (29). The homeostasis model assessment-insulin resistance (HOMA-IR) was determined using the formula [insulin (IU/mL) × FPG (mmol/L)/22.5], following established methodologies (27).

### Statistical analysis

We initially assessed the baseline data distribution by categorizing it into tertiles based on the TyG index. Continuous data were reported as medians with interquartile ranges (25th-75th percentile) or means with standard deviations (SD), while categorical data were expressed as frequencies and percentages. To assess disparities between TyG index groups, The Kruskal-Wallis H test, chi-square test, and one-way ANOVA were employed. Cumulative incidence rates were used to express incidence rates.

The study employed both univariate and multivariate logistic regression to establish three models. Model 1 did not incorporate any covariates, while Model 2 adjusted only for sociodemographic factors, including parity, age, and pre-pregnancy BMI. In contrast, Model 3 encompassed all factors, including parity, age, hepatic steatosis, pre-pregnancy BMI, AST, HDL-C, GGT, LDL-C, insulin, ALT, and TC. Adjusted odds ratios (OR) and their corresponding 95% confidence intervals (CI) were computed to assess GDM risk. Adjustments were made for covariates, and when the inclusion of a covariate in the model resulted in an OR change of at least 10% (30), it was deemed necessary to include that covariate for adjustment.

The current research applied some sensitivity analyses to assess robust results. To assess the relationship of the TyG index as a continuous variable and explore potential non-linearity, we categorized the TyG index into tertiles and calculated the *p* value for trend. The presence of obesity and nonalcoholic fatty liver disease was connected to GDM risk (31, 32). In other sensitivity analyses, we excluded individuals with a grade of hepatic steatosis >0 or pre-pregnancy BMI ≥ 25 kg/m^2^ to assess the connection between the TyG index and GDM. The present study employed a generalized additive model (GAM) to incorporate the continuity variables into the equation as a curve to examine the robustness of our findings (Model 4) (33). Furthermore, we computed E-values to evaluate the potential impact of unmeasured confounding between the TyG index and GDM (34).

Moreover, we applied the stratified logistic regression model to the subgroup analysis, including HOMA-IR, hepatic steatosis, pre-pregnancy BMI, age, and parity. Initially, continuous variables such as HOMA-IR (≤2, >2), pre-pregnancy BMI (<25, ≥25 kg/m^2^), and age (<35, ≥35 years) were discretized according to clinical cutoff points. Subsequently, apart from the stratification factor, we introduced adjustments for all variables (parity, age, hepatic steatosis, pre-pregnancy BMI, AST, HDL-C, GGT, LDL-C, insulin, ALT, and TC) within each stratification. To validate interactions among subgroups, we executed a likelihood ratio test.

Moreover, we conducted a receiver operating characteristic (ROC) analysis to assess the predictive capacity of the TyG index for GDM. The area under the curve (AUC) of the ROC and the optimal threshold were calculated. For all results, the STROBE declaration was followed (30). R software version 3.6 and EmpowerStats (R) version 4.0 were used for all statistical analyses. *P-*values of 0.05 were used to determine statistical significance.

## Results

### Characteristics of participants

This study involved 589 pregnant women with no previous diagnosis of GDM. The average age of the participants was 32.065 ± 3.798 years. The mean TyG index was 8.352 ± 0.400. Between the 24th and 28th weeks of pregnancy, 36 (6.112%) women experienced GDM.

Table 1 lists the baseline characteristics of the pregnant women. Based on the tertiles of the TyG index values, the individuals were split into three groups (T1 ≤ 8.181; 8.181 < T2 ≤ 8.514; T3 > 8.514). It was shown that individuals in the T3 group tended to be older, have higher LDL-C, insulin, GGT, TC, FPG, TG, pre-pregnancy BMI, and a lower prevalence of grade 0 hepatic steatosis.

**Table 1:** The baseline characteristics of participants.

TyG index	T1 (≤8.181)	T2 (8.181–≤8.514)	T3 (>8.514)	*P*-value
Participants	196	196	197	
Age(years)	31.612 ± 3.591	31.888 ± 3.638	32.690 ± 4.081	0.014
Pre-pregnancy BMI (kg/m^2^)	21.026 ± 2.757	21.765 ± 3.491	23.265 ± 3.758	<0.001
Parity				0.072
No	116 (59.184%)	99 (50.510%)	95 (48.223%)	
Yes	80 (40.816%)	97 (49.490%)	102 (51.777%)	
Hepatic steatosis				<0.001
Grade 0	171 (87.245%)	169 (86.224%)	139 (70.558%)	
Grade 1	25 (12.755%)	22 (11.224%)	38 (19.289%)	
Grade 2	0 (0.000%)	4 (2.041%)	13 (6.599%)	
Grade 3	0 (0.000%)	1 (0.510%)	7 (3.553%)	
HDL-C (mg/dL)	66.210 ± 12.574	65.372 ± 13.805	63.119 ± 14.079	0.064
TG (mg/dL)	77.852 ± 14.441	111.061 ± 15.868	167.503 ± 46.743	<0.001
TC (mg/dL)	161.138 ± 21.966	173.469 ± 24.963	183.756 ± 29.325	<0.001
LDL-C (mg/dL)	79.357 ± 18.486	85.518 ± 20.407	87.135 ± 25.225	<0.001
ALT (IU/L)	11 (8–13.25)	11 (8–15)	12 (8–18)	0.095
AST (IU/L)	16 (14–18.25)	16 (14–19)	17 (14–21)	0.161
GGT(IU/L)	11 (10–14)	11 (10–15)	13 (10–18)	0.002
FPG (mg/dL)	73.260 ± 8.010	76.847 ± 8.850	80.964 ± 10.600	<0.001
Insulin (μIU/mL)	7.172 ± 4.030	8.725 ± 4.363	12.646 ± 8.987	<0.001

Values were n(%) or mean ± SD or median (quartile). TyG index, triglyceride-glucose index; BMI: body mass index; ALT, alanine aminotransferase; AST, aspartate aminotransferase; GGT, gamma-glutamyl transferase; HDL-C, high-density lipoprotein cholesterol; TC, total cholesterol; TG, triglycerides; LDL-C, low-density lipid cholesterol; FPG, fasting plasma glucose.

### The prevalence rate of GDM

Table 2 displays the prevalence rate of GDM. Specifically, the prevalence rates of GDM were 6.112% (4.172–8.052%), 1.020% (−0.399–2.440%), 3.061% (0.628–5.494%), and 14.213% (9.294–19.132%) for the overall population of women and for the three TyG index groups (T1groups, T2groups, T3groups). Participants in T3 exhibited a significantly higher prevalence rate of GDM than those in the T1 group (*p* < 0.001 for trend).

**Table 2:** Incidence rate of incident gestational diabetes mellitus.

TyG index	Participants (*n*)	GDM events (*n*)	Cumulative incidence rate (95% CI) (%)
Total	589	36	6.112 (4.172–8.052)
T1	196	2	1.020 (−0.399–2.440)
T2	196	6	3.061 (0.628–5.494)
T3	197	28	14.213 (9.294–19.132)
P for trend			<0.001

TyG index, triglyceride-glucose index; CI, confidence interval; GDM, gestational diabetes mellitus.

### The results of univariate analyses

The outcomes of the univariate analysis have been presented in Table 3. The univariate analysis results indicate that pre-pregnancy BMI, TG, grade of hepatic steatosis, insulin, GGT, FPG, TyG index, and ALT were positively correlated with the occurrence of GDM. Additionally, an inverse association was observed between HDL-C and incident GDM.

**Table 3:** The results of the univariate analysis.

	Statistics	OR (95% CI)	*P-*value
Participants
Age (years)	32.065 ± 3.798	1.037 (0.949, 1.134)	0.42325
Pre-pregnancy BMI (kg/m^2^)	22.019 ± 3.483	1.275 (1.175, 1.384)	<0.00001
Parity
No	310 (52.632%)	ref	
Yes	279 (47.368%)	0.994 (0.506, 1.952)	0.98553
Hepatic steatosis
Grade 0	479 (81.324%)	ref	
Grade 1	85 (14.431%)	3.427 (1.462, 8.033)	0.00460
Grade 2	17 (2.886%)	25.722 (8.780, 75.359)	<0.00001
Grade 3	8 (1.358%)	17.362 (3.814, 79.042)	0.00022
HDL-C (mg/dL)	64.897 ± 13.543	0.964 (0.938, 0.989)	0.00602
TG (mg/dL)	118.888 ± 47.482	1.018 (1.012, 1.025)	<0.00001
TC (mg/dL)	172.806 ± 27.185	1.010 (0.998, 1.022)	0.09210
LDL-C (mg/dL)	84.009 ± 21.789	1.000 (0.985, 1.016)	0.99692
ALT (IU/L)	13.414 ± 9.587	1.037 (1.014, 1.061)	0.00172
AST (IU/L)	17.802 ± 8.101	1.019 (0.992, 1.046)	0.17401
GGT(IU/L)	13.963 ± 8.455	1.034 (1.008, 1.062)	0.01130
FPG (mg/dL)	77.031 ± 9.728	1.069 (1.037, 1.103)	0.00002
Insulin (μIU/mL)	9.524 ± 6.632	1.116 (1.066, 1.169)	<0.00001
TyG index	8.352 ± 0.400	30.230 (10.535, 86.746)	<0.00001

Values are n(%) or mean ± SD. TyG index, triglyceride-glucose index; BMI, body mass index; ALT, alanine aminotransferase; AST, aspartate aminotransferase; GGT, gamma-glutamyl transferase; HDL-C, high-density lipoprotein cholesterol; TC, total cholesterol; TG, triglycerides; LDL-C, low-density lipid cholesterol; FPG, fasting plasma glucose.

### The results of multivariate analyses

Table 4 demonstrates the application of a multivariate logistic regression model to explore the link between the TyG index and incident GDM. In Model 1, a positive connection was observed between the TyG index and incident GDM (OR: 30.230, 95%CI: 10.535–86.746, *p* < 0.00001). Model 2, which incorporated adjustments for age, pre-pregnancy BMI, and parity, yielded consistent outcomes with no significant alterations (OR: 17.816, 95%CI: 5.511–57.588, p < 0.00001). Moreover, even after accounting for variables including parity, age, hepatic steatosis, pre-pregnancy BMI, AST, HDL-C, GGT, LDL-C, insulin, ALT, and TC in Model 3, a noticeable connection between the TyG index and incident GDM persisted (OR: 12.923, 95%CI: 3.581–46.632, *p* = 0.00009). These findings imply that a 12-fold increase in the likelihood of getting GDM is associated with each unit rise in the TyG index.

**Table 4:** Relationship between TyG index and the incident GDM in different models.

Variable	Model 1 (OR.,95% CI, P)	Model 2 (OR,95% CI, P)	Model 3 (OR, 95% CI, P)	Model 4 (OR, 95% CI, P)
TyG index	30.230 (10.535, 86.746) <0.00001	17.816 (5.511, 57.588) <0.00001	12.923 (3.581, 46.632) 0.00009	19.836 (4.699, 83.743) 0.00005
TyG index (tertile)
T1	Ref	Ref	Ref	1.0
T2	3.063 (0.611, 15.367) 0.17369	2.276 (0.435, 11.900) 0.32978	1.811 (0.342, 9.606) 0.48520	1.222 (0.210, 7.105) 0.82297
T3	16.071 (3.772, 68.465) 0.00017	8.543 (1.913, 38.153) 0.00496	5.618 (1.194, 26.438) 0.02896	5.586 (1.159, 26.922) 0.03202
P for trend	<0.00001	0.00056	0.00687	0.00520

Model 1: we did not adjust other covariants. Model 2: we adjusted age, pre-pregnancy BMI, parity. Model 3: we adjusted age, pre-pregnancy BMI, parity, hepatic steatosis, AST, GGT, ALT, TC, HDL-C, LDL-C, insulin. Model 4: we adjusted age(smooth), pre-pregnancy BMI(smooth), parity, hepatic steatosis, AST (smooth), GGT (smooth), ALT (smooth), TC (smooth), TC (smooth), HDL-C (smooth), insulin (smooth). OR, odds ratios; CI, confidence interval; Ref, Reference; TyG index, triglyceride-glucose index.

### Sensitive analysis

We reintroduced the TyG index after categorically transforming it from a continuous variable. Compared to the reference category (T1) of the TyG index, the multivariate-adjusted model exhibited an HR of 1.811 (95%CI: 0.342–9.606) in the T2 group and 5.618 (95% CI: 1.194–26.438) in the T3 group (Table 4).

The continuity covariate was introduced into the equation as a curve using a GAM. According to the results of model 4, the TyG index is positively correlated with the risk of GDM (HR:19.836，95%CI: 4.699–83.743) (Table 4). Notably, the E value for this study was 25.34, surpassing the relative risk of the TyG index and potential unmeasured confounders. This outcome suggested that the association between the TyG index and incident GDM remained largely unaffected by unmeasured or unknown confounders.

Furthermore, we conducted sensitivity analyses on subjects with BMI < 25 kg/m^2^. The TyG index was found to be positively correlated with the risk of GDM after adjusting for parity, age, hepatic steatosis, pre-pregnancy BMI, AST, HDL-C, GGT, LDL-C, insulin, ALT, and TC (OR: 13.204, 95%CI: 2.547–68.446, *p* = 0.00211) (Table 5). Similarly, even when individuals with grade 0 hepatic steatosis were included in additional sensitivity analyses, the positive relationship between the TyG index and the likelihood of developing GDM persisted after adjusting for confounding covariates (OR: 10.524, 95%CI: 1.925–57.547, *p* = 0.00662) (Table 5). The sensitivity analysis supported the robustness of our conclusions. Notably, the E value for this study was 25.34, surpassing the relative risk of the TyG index and potential unmeasured confounders. This outcome suggested that the connection between the TyG index and GDM risk remained largely unaffected by unmeasured or unknown confounders.

**Table 5:** Relationship between TyG index and incident GDM in different sensitivity analyses.

Exposure	Model 5 (OR, 95%CI, P)	Model 6 (OR, 95%CI, P)
TyG index	13.204 (2.547, 68.446) 0.00211	10.524 (1.925, 57.547) 0.00662
TyG index (tertile)
Q1	Ref	Ref
Q2	1.916 (0.342, 10.720) 0.45939	1.498 (0.256, 8.768) 0.65374
Q3	3.579 (0.666, 19.235) 0.13722	4.221 (0.809, 22.023) 0.08749
P for trend	0.11446	0.04806

Model 5 was sensitivity analysis after excluding those with pre-pregnancy BMI ≥ 25 kg/m*^2^*. We adjusted age, pre-pregnancy BMI, parity, hepatic steatosis, AST, GGT, ALT, TC, HDL-C, LDL-C, insulin. Model 6 was sensitivity analysis after including those with grade 0 hepatic steatosis. We adjusted age, pre-pregnancy BMI, parity, AST, GGT, ALT, TC, HDL-C, LDL-C, insulin. OR, odds ratios; CI, confidence; Ref: reference; TyG index, triglyceride-glucose index.

### The results of the subgroup analysis

The connection between the TyG index and GDM risk was examined using subgroup analysis (Table 6) to find potential confounding factors that may have impacted the results. HOMA-IR, parity, pre-pregnancy BMI, hepatic steatosis, and age were selected as stratification factors. It was determined that those mentioned above potential confounding variables did not impact the association between the TyG index and the risk of GDM. The results of the subgroup analysis underscore the robustness of our conclusions.

**Table 6:** Effect size of TyG index on GDM in prespecified and exploratory subgroups.

Characteristic	No of patients	Effect size (95%CI)	*P-*value	*P* for interaction
Age (years)				0.9935
<35	452	21.926 (4.544, 105.803)	0.0001	
≥35	137	22.256 (0.881, 562.024)	0.0597	
Pre-pregnancy BMI (kg/m^2^)				0.3923
<25	493	11.807 (2.559, 54.487)	0.0016	
≥25	95	41.965 (2.979, 591.166)	0.0056	
Parity				0.3476
No	310	24.258 (3.849, 152.869)	0.0007	
Yes	279	7.246 (1.162, 45.207)	0.0340	
Hepatic steatosis				0.8196
Grade 0	479	10.765 (2.025, 57.236)	0.0053	
Grade 1–3	110	14.552 (1.965, 107.793)	0.0088	
HOMA-IR				0.5168
≤2	388	9.991 (1.484, 67.258)	0.0180	
>2	201	22.912 (4.235, 123.956)	0.0003	

Above model adjusted for we adjusted for age, pre-pregnancy BMI, parity, hepatic steatosis, AST, GGT, ALT, TC, HDL-C, LDL-C, insulin. The model is not adjusted for the stratification variable in each case.

### ROC analysis

**Figure 2:**
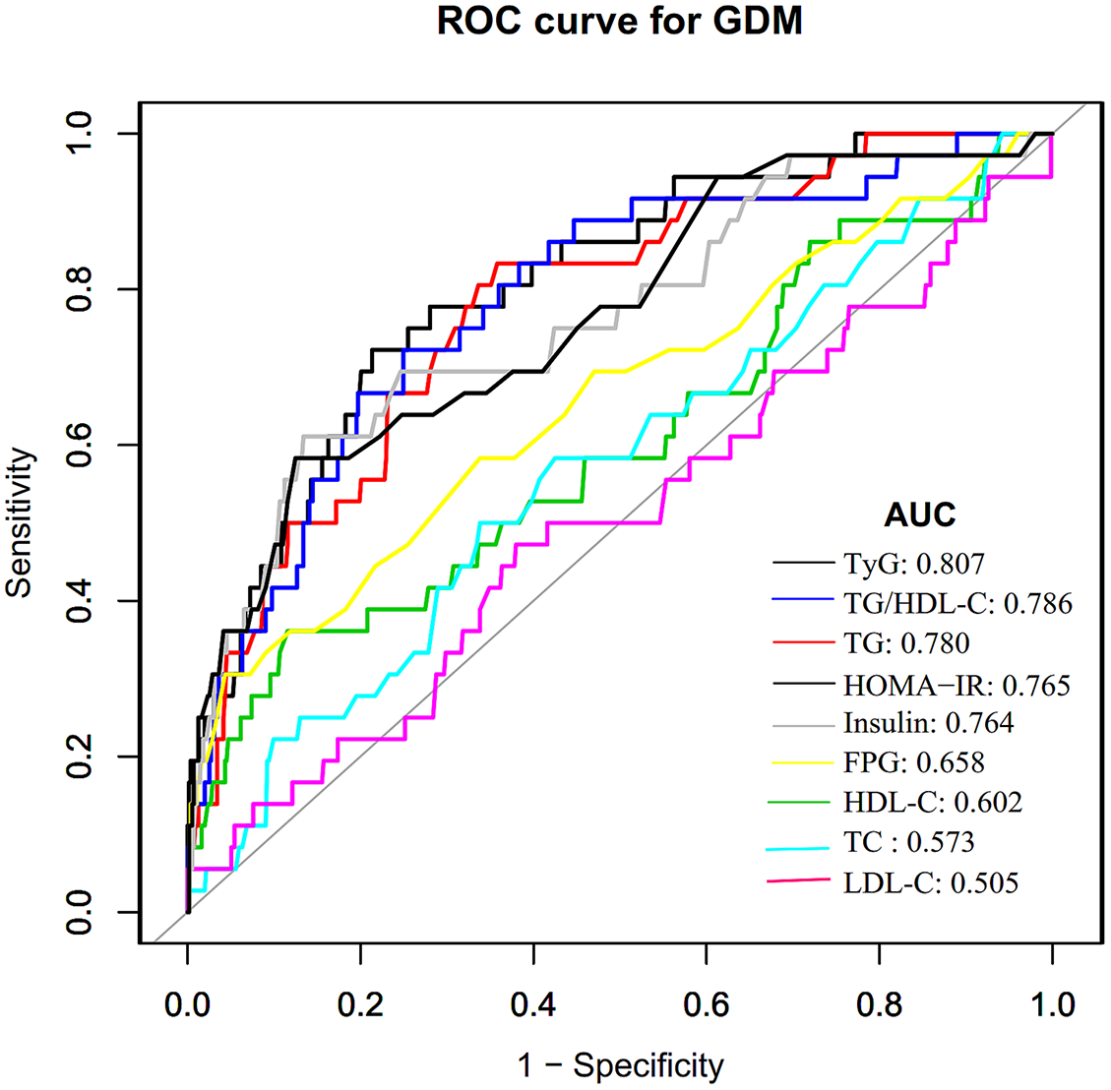
The TyG index for predicting DM in all participants by ROC analyses. ROC analysis was further conducted to explore the ability of the TyG index to predict GDM. The results showed that the AUC of the TyG index was 0.807. Compared to TG, HDL-C, TG/HDL-C ratio, TC, LDL-C, FPG, insulin, and HOMA-IR, the AUC of the TyG index for predicting DM was the highest.

ROC analysis was performed to assess the predictive capacity of the TyG index for GDM. The results revealed an AUC of 0.807 (95% CI: 0.734–0.879), as presented in Table 7 and Figure 2. In comparison to other factors, including TG, triglyceride to high-density lipoprotein cholesterol ratio (TG/HDL-C), FPG, HDL-C, HOMA-IR, TC, insulin, and LDL-C, the TyG index exhibited the highest AUC for GDM prediction. Using Youden’s index, the optimal cutoff point for the TyG index to predict GDM was determined to be 8.632. This threshold corresponded to a specificity of 78.7% and a sensitivity of 72.2%.

**Table 7:** Areas under the receiver operating characteristic curves (AUROC) for each evaluated parameters in identifying GDM.

Test	AUROC	95%CI	Best threshold	Specificity	Sensitivity	Youden Index
TyG index	0.807	0.734–0.879	8.632	0.787	0.722	0.509
TG	0.780	0.704–0.856	121.500	0.642	0.833	0.475
HDL-C	0.602	0.496–0.709	49.200	0.884	0.361	0.245
TG/HDL-C ratio	0.786	0.707–0.866	2.268	0.751	0.722	0.473
TC	0.573	0.473–0.672	181.500	0.662	0.500	0.162
LDL-C	0.505	0.400–0.611	77.550	0.620	0.472	0.092
FPG	0.658	0.555–0.762	90.500	0.957	0.306	0.263
Insulin	0.764	0.675–0.853	13.900	0.866	0.611	0.477
HOMA-IR	0.765	0.679–0.851	2.7500	0.875	0.583	0.458

## Discussion

This study explored the relationship between the TyG index and GDM risk within the Korean population. Our findings unveiled a positive connection between the TyG index and incident GDM. Notably, a 12-fold increase in the likelihood of getting GDM is associated with each unit rise in the TyG index. Our findings revealed a higher diagnostic efficiency with an AUC of 0.807 (95%CI: 0.734–0.879) for the TyG index in predicting GDM, which is significantly superior to the AUC values reported in similar studies, ranging from 0.57 to 0.69 (25, 26, 35). These similar studies rely on FPG, a one-step 75 g OGTT, or self-reported diagnosis of GDM. The use of a two-step testing procedure for GDM diagnosis in our study may have contributed to the higher diagnostic accuracy of the TyG index. In addition, this disparity in diagnostic performance may also be related to study design, population characteristics, and sample size.

The prevalence of GDM has seen an uptick to 12.70% within the broader Korean population in recent times (36). Interestingly, the prevalence of GDM within the scope of this study was found to be 6.112%, which is comparatively lower than the documented rates. This current study used stricter exclusion criteria (excessive alcohol consumption, chronic liver disease, or previous diagnosis of GDM) as well as diagnostic criteria for GDM (using the two-step test), all of which would have led to a decrease in the prevalence of GDM in this current study. Consequently, the lower GDM incidence among research participants finds validation within this context. However, it’s worth highlighting that the GDM prevalence still stands at 6.112% within this population. This emphasizes the continued importance of exploring potential additional risk factors for GDM.

Impaired insulin sensitivity or insulin secretion is widely recognized as the main underlying pathology of gestational diabetes mellitus. Women with dominant insulin resistance and GDM are more likely to experience negative effects. Conventional indicators of IR, such as the euglycemic-hyperinsulinemic clamp, face limitations due to invasiveness and complexity in clinical settings. Accessibility issues and a lack of clear cutoff values additionally hamper these techniques. Additionally, GDM is often detected between 24 and 28 weeks of pregnancy, giving little opportunity to prevent it from developing and causing damage. Thus, it becomes imperative to identify women susceptible to GDM early in pregnancy, aiming to reduce its impact using a proxy marker of insulin resistance. According to several findings, the TyG index could serve as a valuable indicator of insulin resistance. It has demonstrated potential in foretelling the beginning and development of diabetes in the general population. In two separate studies involving 352 Chinese women and 954 Iranian (23, 26), those in the highest tertile of the first-trimester TyG index were found to be 3.54-fold and 4.91-fold more likely to develop GDM, respectively. The Korean National Health Screening Exam study further highlights that an increase in the TyG index of just one standard deviation 2 years before conception increases the risk of gestational diabetes by 33% (25). A subsequent meta-analysis has confirmed and reinforced these findings (37). This study emphasizes that the risk of confirmed GDM within the Korean population increases with a rising TyG index, even after accounting for confounding variables. In our sensitivity analysis, we observed that the connection between the TyG index and GDM risk remains significant among Korean women with a BMI of less than 25 kg/m^2^or with grade 0 hereditary steatosis. Moreover, we expanded our adjustments to include additional covariates like insulin, AST, hepatic steatosis, and GGT, which are all recognized risk factors for GDM (2, 32). Further analyses stratified by HOMA-IR, parity, pre-pregnancy BMI, hepatic steatosis, and age yielded consistent results, underscoring the stability of the relationship between the TyG and GDM risk. Consequently, this study broadens the applicability of the association between the TyG and GDM to the wider population. As such, this research holds substantial clinical significance. The implications of this study may serve as a stepping stone for future endeavors in developing predictive models for GDM.

The predictive capacity of the TyG index for GDM or T2DM has been extensively investigated, with consistent threshold values found across various studies (24–26, 35, 38, 39). Notably, Wang et al. (38) conducted a 15-year prospective study in Chinese adults, revealing a threshold of around 8.51 for the TyG index’s impact on incident T2DM risk. Similarly, Lee et al. (40) established a TyG index cutoff of 8.52 for predicting T2DM in more than 7,000 Korean adults. Kim et al. (25) reported a TyG index cutoff of 8.15 (AUC 0.60, specificity 68.2%, sensitivity 47.0%) for forecasting GDM 2 years before pregnancy. Regarding the diagnostic performance of the TyG index in detecting GDM during pregnancy, an AUC of 0.686 (95%CI: 0.615–0.756) was obtained by Liu et al. (26) in their evaluation of the TyG index’s diagnostic capacity to predict GDM during the first prenatal visit. At the same time, no specific threshold value was specified. Similarly, Sanchez-Garcia et al. (39) identified a relatively low cutoff value of 4.69 (specificity 50%, sensitivity 89.0%). In addition, Zeng Y et al. found limited diagnostic efficacy of the TyG index for GDM (AUC = 0.57, 95% CI: 0.50–0.63) (35). In the current study, the TyG index demonstrated robust predictive capability for GDM, with an AUC of 80.7% and an optimal predictive cutoff value of around 8.632. Furthermore, the TyG index outperformed TG, HDL-C, TG/HDL-C, TC, LDL-C, FPG, insulin, and HOMA-IR indices in predicting GDM. Remarkably, the TyG index’s diagnostic accuracy in GDM surpassed that of the HOMA-IR, suggesting its potential as an early biomarker for insulin resistance in early pregnancy and a reliable indicator for GDM detection.

However, the mechanism by which TyG associates with GDM is unclear. Firstly, the TyG index is a useful marker for insulin resistance (41–43), a core pathophysiological feature of GDM. Insulin resistance leads to reduced glucose uptake by peripheral tissues and increased hepatic glucose production, contributing to hyperglycemia during pregnancy (44). Secondly, high triglyceride levels, as part of the TyG index, suggest a disturbance in lipid metabolism as a consequence of hyperglycemia. This dyslipidemia can lead to an accumulation of fatty acids in tissues such as muscle and liver, which can interfere with insulin signaling and exacerbate insulin resistance, thereby increasing the risk of GDM (35). Furthermore, FPG levels reflect insulin sensitivity of the liver and insulin secretion by pancreatic β-cells, which are key factors in the pathogenesis of GDM (2, 32). Thus, the underlying mechanism of the TyG index’s association with GDM risk can be attributed to the interplay between FPG and TG, both of which are associated with insulin resistance.

Our study presents several notable strengths. Firstly, we utilized tertiles of the TyG index as a categorical and continuous variable in our independent variables, enabling a comprehensive examination of its association with GDM risk. Secondly, meticulous statistical adjustments were employed to minimize the impact of residual confounding factors. Thirdly, subgroup analyses were conducted to evaluate the influence of other potential risk factors on the relationship between the TyG index and GDM.

However, certain limitations of our study should be acknowledged. Firstly, the association between the TyG index and GDM might exhibit variations across different ethnicities, underscoring the need for validation in diverse ethnic groups. Secondly, as a secondary analysis, our research could not adjust for variables like uric acid, hypertension, and renal function, which were not originally present in the dataset. Thirdly, the original study did not account for the fluctuations in FPG and TG over time. As previously reported (45), serum triglycerides are increased 2–3 times by late pregnancy, although they progressively increase from the first phases. Besides, triglycerides are subject to considerable analytical variability and, to an even greater extent, biological variability, exhibiting fluctuations that may range between 20 and 40% (46, 47). Future iterations of our investigation could encompass these additional confounding variables and track changes in FPG and TG throughout the follow-up period. Fourthly, there may be an impact on the results due to the existence of intra-coefficient variation and inter-coefficient variation for TG and FPG. In the future, we can consider designing our study with multiple measurements of TG and FPG on the same specimen to avoid influencing our results.

## Conclusion

In conclusion, this study underscores the independent and positive correlation between an elevated TyG index and the risk of developing incident GDM within the Korean population. As such, the abnormal TyG index could be a valuable predictor for GDM. Consequently, it aids in identifying individuals in Korea who are at a heightened risk of GDM development. This finding holds the potential to aid healthcare practitioners in formulating and applying effective care strategies. Additionally, it might function as an early screening and monitoring tool to curtail the onset and advancement of GDM within clinical settings.

## Data availability statement

The datasets presented in this study can be found in online repositories. The names of the repository/repositories and accession number(s) can be found here: https://journals.plos.org/plosone/article?id=10.1371/journal.pone.0221400.

## Ethics statement

The studies involving humans were approved by the committee of the Seoul Metropolitan Government Seoul National University Boramae Medical Center and the committee of the Ministry of Health and Welfare of Korea. The studies were conducted in accordance with the local legislation and institutional requirements. The participants provided their written informed consent to participate in this study. Written informed consent was obtained from the individual(s) for the publication of any potentially identifiable images or data included in this article.

## Author contributions

ZM: Writing – original draft. CC: Supervision, Writing – original draft. YHa: Writing – original draft. HH: Writing – review & editing. YHe: Writing – review & editing. XZ: Writing – review & editing.
